# Electronic cigarette use among adolescents in Saudi Arabia: A national study, 2022

**DOI:** 10.18332/tid/197410

**Published:** 2025-01-21

**Authors:** Shaikha K. AlDukhail, Eman D. El Desouky, Sarah S. Monshi, Abdulmohsen H. Al-Zalabani, Abdullah M. Alanazi, Mervat M. El Dalatony, Mosa A. Shubayr, Ahmed A. Elkhobby, Mohammed M. Alqahtani, Anwar S. Alhazmi, Mohammed S. Aldossary

**Affiliations:** 1 Department of Preventive Dental Sciences, College of Dentistry, Princess Nourah bint Abdulrahman University, Riyadh, Saudi Arabia; 2 General Directorate of Research and Studies, Ministry of Health, Riyadh, Saudi Arabia; 3 Department of Health Administration and Hospitals, College of Public Health and Health Informatics, Umm Al-Qura University, Makkah, Saudi Arabia; 4 Department of Family and Community Medicine, College of Medicine, Taibah University, Madinah, Saudi Arabia; 5 Department of Respiratory Therapy, College of Applied Medical Sciences, King Saud bin Abdulaziz University for Health Sciences, Riyadh, Saudi Arabia; 6 King Abdullah International Medical Research Center, Riyadh, Saudi Arabia; 7 Department of Respiratory Services, King Abdulaziz Medical City, Ministry of National Guard Health Affairs, Riyadh, Saudi Arabia; 8 Public Health and Community Medicine Department, Faculty of Medicine, Menoufia University, Egypt; 9 Department of Preventive Dental Sciences, College of Dentistry, Jazan University, Jazan, Saudi Arabia; 10 Tobacco Control Program, Ministry of Health, Riyadh, Saudi Arabia

**Keywords:** e-cigarette, electronic nicotine delivery systems, adolescent health

## Abstract

**INTRODUCTION:**

Electronic cigarette (e-cigarette) use has increased globally among adolescents. However, data on its use among adolescents in Saudi Arabia remain limited. Therefore, this study describes the characteristics and factors associated with e-cigarette use in this population.

**METHODS:**

Data from the Saudi Arabia 2022 Global Youth Tobacco Survey targeting students aged 13–15 years were analyzed. This study examined current use and ever use of e-cigarettes as dependent variables, and sociodemographic characteristics, smoking behaviors, and exposure to tobacco-related messages as independent variables.

**RESULTS:**

Among the adolescents, 14.2% had ever used e-cigarettes, while 5.3% were current users. Older adolescents exhibited a higher prevalence of ever use (12%, 13%, and 19% among those aged 13, 14, and 15 years, respectively). Of those who ever smoked cigarettes, 44% reported ever using e-cigarettes, and 23% were current users. Older adolescents (15 years) were 35% more likely to be ever e-cigarette users (AOR=1.35; 95% CI: 1.32–1.38) and 39% more likely to be current e-cigarette users (AOR=1.39; 95% CI: 1.34–1.43) than those aged 13 years. Exposure to pro-tobacco marketing was associated with twice the odds of ever using e-cigarettes (AOR=2.23; 95% CI: 2.21–2.26) and 2.76 times the odds of becoming current e-cigarette users (AOR=2.64; 95% CI: 2.58–2.69) compared with non-exposed adolescents.

**CONCLUSIONS:**

E-cigarette use is more prevalent among older adolescents, males, and those with higher weekly allowances. Additionally, e-cigarette use is associated with adolescents who use tobacco cigarettes, are exposed to secondhand smoke, or encounter tobacco promotional marketing.

## INTRODUCTION

Adolescence (age 10–19 years)^1^, is critical for physical and mental growth. The neurocognitive and hormonal changes inherent to adolescence are consistent developmental factors. Nevertheless, these biological transformations unfold within the broader socio-ecological frameworks encompassing social and environmental factors influencing their behaviors^2^. This population is more vulnerable to the risks of addiction, including tobacco use. Globally, one in every ten adolescents engages in tobacco use, and its prevalence rate varies across different countries^3^. Tobacco use by adolescents has adverse consequences, such as premature atherosclerosis, diminished lung function, complications in oral health, impulse control difficulties, and reduced physical endurance^4,5^. These consequences contribute to increasing the government’s economic burden resulting from premature death, illnesses, and productivity loss caused by school absenteeism, and physical and mental impairment^6^.

Saudi Arabia is one of the countries confronted with the pressing concern of tobacco use. Various forms of tobacco products are used, including conventional cigarettes, waterpipe (shisha), smokeless tobacco, and electronic nicotine delivery systems (ENDS), such as electronic cigarettes (e-cigarettes) and vapes^7^. The current prevalence of tobacco use among adults is 19.8% (male, 30%; female, 4.2%)^7^. Furthermore, tobacco use generally begins before reaching the age of 18 years^8^, thereby increasing the risk of dependence and diminishing the likelihood of successful quitting^9^. Despite a decrease in its prevalence among adolescents in Saudi Arabia from 15.9% (male, 20.2%; female, 10.7%) in 2007 to 9.4% (male, 10.6%; female, 8%) in 2022^10^, the problem continues to exist in this age group, considering the emergence of novel nicotine and tobacco products.

E-cigarettes have emerged as one of the predominant tobacco products among youth. The escalating prevalence of e-cigarette use is disconcerting. From January 2017 to March 2022 in the United States, the unit share of e-cigarettes containing ≥5% nicotine strength substantially surged to 1486.3%, whereas the dollar share notably increased to 1345.5%^11^. In Saudi Arabia, recent research has revealed that the prevalence of e-cigarette use among students in various health colleges reached 32.36%, while that among school students was 5.4%^10^. The issue worsens as several studies have consistently established a positive association between e-cigarette use and the initiation of other tobacco products use (e.g. e-cigarettes and conventional cigarettes)^12^.

Several factors could influence e-cigarette use among youth. At the individual level, personal characteristics, social norms, and peer influence strongly affect e-cigarette initiation and its continued use^13^. If youths perceive e-cigarette use as socially acceptable, they are more likely to adopt it^14^. Additionally, parental attitudes and behaviors toward tobacco use can either discourage or unintentionally encourage its use among youths^15^. At the broader level, the widespread e-cigarette availability in retail stores^16^, with extensive marketing and promotion^17^, also contributes to e-cigarette experimentation and uptake in this age group.

Despite that earlier investigations have underscored e-cigarettes’ adverse health effects on children and young individuals, particularly those who never use tobacco^12^, the tobacco industry continues to promote e-cigarettes as an eco-friendly and safer alternative^17^. Moreover, these companies target vulnerable populations, including minors^18,19^, through direct advertising, free samples, and indirect strategies such as event sponsorship and tobacco imagery placement in media^18^. Data further illuminate the extent of these practices, whereby e-cigarette companies, mostly tobacco companies, have spent approximately $859.4 million in advertising expenditures^20^.

The cultural and regulatory landscape shapes adolescent behaviors around tobacco and e-cigarette use in Saudi Arabia. Smoking, once stigmatized – especially for women – has become more accepted among younger people. Media advertising of e-cigarettes has further normalized their use across demographics, including youth and women^7,8,10,21^. Saudi Arabia, a signatory to the WHO Framework Convention on Tobacco Control (FCTC), has established comprehensive tobacco policies. National laws ban smoking in many public places, prohibit tobacco cultivation, restrict sales to minors, and offer cessation support. While the import ban on e-cigarettes was lifted in 2019, their use is still regulated under existing tobacco laws. However, ambiguity remains in the regulation of e-cigarette advertising and labelling, complicating the understanding of their influence on adolescent use^7,8,10,21^.

E-cigarette use among adolescents has risen globally, but data on its prevalence in Saudi Arabia remains limited. Existing studies often rely on small, non-representative surveys, hindering accurate conclusions about the scope and factors behind adolescent use. These limitations also make it difficult to compare Saudi trends with other nations and develop effective public health strategies. The Global Youth Tobacco Survey (GYTS) offers a nationally representative dataset that can address this gap. This study used the recent GYTS data to assess the prevalence, characteristics, and associated factors of e-cigarette use among Saudi adolescents.

## METHODS

### Study population, design, and setting

In Saudi Arabia, the GYTS was conducted jointly by the Ministry of Health and the Ministry of Education in 2022. The GYTS is a nationally representative, cross-sectional school-based survey targeting students aged 13–15 years (grades 1–3 of intermediate schools).

GYTS employs a globally standardized methodology^22,23^, utilizing a two-stage sample design wherein schools are selected according to probability proportional to the enrollment size. Classes within selected schools are then chosen randomly, with all students in these classes eligible to participate in the survey^23^. In Saudi Arabia, sampling and weighting were conducted separately for two strata (males and females). Subsequently, a national dataset was compiled by merging data files from all regions in the country.

The survey uses a standard core questionnaire with optional questions that countries can adapt to measure and track key tobacco control indicators. The questionnaire covers the following topics: tobacco use (smoking and smokeless), cessation, secondhand smoke (SHS), pro- and anti-tobacco advertising and promotion, access to and availability of tobacco products, and knowledge and attitudes regarding tobacco use. The questionnaire is self-administered, maintaining anonymity by using scannable paper-based bubble sheets to ensure confidential^23^.

### Ethical considerations

This study obtained approval from the Ministry of Education and the principals of the selected schools. Before data collection, parental consent was acquired. Trained personnel from both the Saudi Ministry of Health and Ministry of Education provided instructions on how to fill in the survey and supervise the data collection process. Students were informed that participation was voluntary and that they could withdraw at any time. They could also opt to decline answering any questions they did not wish to answer. The collected data were securely stored with access limited only to personnel involved in its analysis. Institutional review board approval was obtained from the Ministry of Health (protocol number: IRB:23-106 M).

### Measures

Dependent variables included current e-cigarette smoking, which ‘refers to adolescents who have used e-cigarettes on at least one day within the past 30 days prior to the survey’, and ever e-cigarette smoking, which ‘refers to adolescents who have tried or experimented with e-cigarettes at least once in their lifetime, regardless of whether they currently use them’. Independent variables encompassed sociodemographic characteristics, weekly pocket money in SAR (100 Saudi Arabian Riyals about US$27), ever cigarette smoking, exposure to family SHS, exposure to tobacco promotional marketing, and exposure to anti-tobacco messages. We coded exposure to school anti-tobacco messages separately because it represents a specific context within the broader framework of anti-tobacco messaging and may have different influences and effects on youth behavior.

### Statistical analysis

Data were analyzed using Statistical Package for the Social Sciences (IBM SPSS Statistics 27.0 Armonk, NY, USA), incorporating weights and clusters to accommodate the GYTS’ complex multistage survey design, ensuring representative estimates of the Saudi youth population. Categorical data are presented as frequencies and weighted percentages. A weighting factor was applied to account for the varying probabilities of sampling each student and to mitigate bias arising from different patterns of non-response. In univariate analysis, associations between independent variables and current and ever e-cigarette smoking were analyzed using chi-squared test. We also calculated the odds ratio with their corresponding 95% confidence intervals (CIs). In multivariable analysis, independent risk factors for the current and ever e-cigarette smoking were identified through logistic regression analysis. Adjusted odds ratios (AORs) and 95% CIs were then calculated. A value of p<0.05 was considered statistically significant. All tests were two-tailed.

## RESULTS

This study reports the results of 5610 students aged 13–15 years, with an overall response rate of 92.3%.

### Prevalence of e-cigarette use

Among the included adolescents in Saudi Arabia, 14.2% had ever used e-cigarettes, while 5.3% were current users. Older adolescents exhibited a higher prevalence of ever use of e-cigarettes than the younger ones, with 12%, 13%, and 19% among those aged 13, 14, and 15 years, respectively. Similarly, the prevalence of current e-cigarette use increased with age (4%, 4.5%, and 8%, respectively). Males had a higher prevalence of both ever and current e-cigarette use than females. Approximately 40% of adolescents reported exposure to SHS. Exposure was reported at home (18%), enclosed public places (27%), outdoor public places (28%), and inside their school building (22%). Exposure to SHS was associated with a higher prevalence of ever (22.8%) and current (10.4%) e-cigarette use compared with non-exposure (8.5% and 2.0%, respectively). Among those who claimed exposure to SHS at home, 30% reported ever using e-cigarettes, while 15% reported current e-cigarette use (Table 1).

**Table 1:** Demographic characteristic of electronic cigarette users among adolescents in Saudi Arabia, GYTS, 2022 (N=5610)

*Characteristics*	*Categories*	*All* *n (%)*	*Ever users* *n (%)*	*Current users* *n (%)*
**Total**		5610 (100)	791 (14.2)	300 (5.3)
**Age** (years)	13	1928 (34.4)	225 (11.9)	77 (4.0)
	14	2140 (37.1)	278 (13.0)	97 (4.5)
	15	1542 (28.5)	288 (18.5)	126 (8.0)
**Sex**	Male	2941 (50.9)	448 (15.5)	176 (6.0)
	Female	2669 (49.1)	343 (12.9)	124 (4.7)
**Grade**	1st intermediate	1684 (30.8)	211 (12.6)	66 (3.9)
	2nd intermediate	2253 (37.3)	273 (12.1)	107 (4.7)
	3rd intermediate	1673 (31.8)	307 (18.2)	127 (7.5)
**Exposure to secondhand smoke**	No	3343 (59.9)	280 (8.5)	66 (2.0)
	Yes	2253 (40.1)	509 (22.8)	233 (10.4)
**Place of exposure to secondhand tobacco smoke**	Home	1011 (18.3)	306 (30.2)	149 (14.6)
	Enclosed public place	1512 (26.8)	368 (24.7)	175 (11.7)
	Outdoor public place	1597 (28.3)	363 (23.0)	181 (11.4)
	Inside the school building or outside on school property	1216 (22.2)	287 (23.7)	119 (9.8)

All variables are reported with unweighted frequency (n) and weighted percentage (%) from the row total, to account for complex survey design.

### Characteristics and habits of e-cigarette users

Over a third (38.8%) of current e-cigarette users reported first trying e-cigarettes at age 14–15 years, consistent with the ever users (36%). Among those who ever smoked cigarettes, 44% reported ever using e-cigarettes and 23% were currently using e-cigarettes. Regarding access and availability of e-cigarettes, 39% and 29% of ever users reported obtaining it from someone else and a store/shop, respectively. Notably, approximately 43.7% of ever e-cigarette users and 41.6% of current users were refused purchase of e-cigarettes because of their age.

Furthermore, roughly 17.4% of adolescents who were current e-cigarette users reported receiving weekly pocket money ≥100 SAR (Table 2).

**Table 2:** Characteristics and habits of electronic cigarette users among adolescents in Saudi Arabia, GYTS, 2022

*Characteristics*	*Categories*	*N*	*Ever users* *n (%)*	*Current users* *n (%)*
**Total**			791 (14.2)	300 (5.3)
**Age first tried e-cigarettes**	≤7	87	87 (17.9)	50 (19.5)
	8–9	33	33 (6.7)	13 (5.2)
	10–11	36	36 (7.2)	18 (6.7)
	12–13	158	158 (32.2)	78 (29.8)
	14– 15	173	173 (36.0)	99 (38.8)
**Ever smoked cigarettes**	No	4883	473 (60.6)	130 (44.3)
	Yes	727	318 (39.4)	170 (55.7)
**Source of e-cigarettes**	Store/shop	48	-	48 (29.1)
	Street vendor	10	-	10 (6.2)
	Internet	11	-	11 (6.7)
	Someone else	65	-	65 (39.1)
	Other	32	-	32 (18.9)
**Refused purchase of e-cigarettes due to age**	No	87	87 (56.3)	66 (58.4)
	Yes	67	67 (43.7)	47 (41.6)
**Weekly pocket money** (SAR)	None	1266	157 (20.3)	65 (22.2)
	<30	1934	236 (30.3)	62 (21.0)
	30–49	1128	155 (19.8)	56 (18.4)
	50–99	741	131 (16.9)	62 (21.0)
	≥100	491	99 (12.7)	52 (17.4)
Exposure to tobacco promotional marketing*	Not exposed to any marketing	2017	251 (31.7)	74 (24.6)
	Offered a free tobacco product from a tobacco company representative	368	144 (39.5)	66 (17.6)
	Promotions at points of sale	768	170 (22.6)	89 (11.7)
	Television, videos, or movies	2048	347 (17.1)	143 (7.0)
	Had something with a tobacco brand logo on it	489	173 (36.0)	80 (16.7)
Exposure to anti-tobacco messages*	In the media	2370	377 (49.8)	149 (53.4)
	At sporting or community events	1032	160 (42.0)	71 (43.4)
	Were taught in school about the dangers of tobacco use in the past 12 months	2061	307 (39.3)	120 (41.0)

* Can have multiple exposures. All variables are reported with unweighted frequency (n) and weighted percentage (%) to account for complex survey design. SAR: 100 Saudi Arabian Riyals about US$27.

In assessing exposure to tobacco promotional marketing among adolescents in Saudi Arabia, we found that 40% of those who were offered a free tobacco product from a tobacco company representative reported ever using e-cigarettes. Of those exposed to promotions at points of sale, 23% were ever users and 12% were current users.

Anti-tobacco messages the adolescents (current e-cigarette users) were exposed to were mostly via media communications (53.4%), followed by school education about the dangers of tobacco use within the past 12 months (41%) (Table 2).

### Factors associated with e-cigarette use among the adolescents

Multivariable logistic regression analysis revealed several factors significantly associated with ever and current e-cigarette use (Table 3). Older adolescents (15 years) had higher odds of being ever e-cigarette users (AOR=1.35; 95% CI: 1.33–1.38) and current e-cigarette users (AOR=1.39; 95% CI: 1.34–1.43) compared to those aged 13 years. Additionally, males had higher odds of being both ever and current e-cigarette users (AOR=1.37; 95% CI: 1.36–1.39 and AOR=1.42; 95% CI: 1.39–1.45, respectively) compared to females (Table 3).

**Table 3:** Factors associated with electronic cigarette use among adolescents in Saudi Arabia, GYTS, 2022

*Characteristics*	*Categories*	*Ever users* *N=791 (14.2%)*		*Current users* *N=300 (5.3%)*	
		OR (95% CI)*	AOR (95% CI)*	OR (95% CI)*	AOR (95% CI)*
**Age** (years)	13 ®	1	1	1	1
	14	1.11 (1.09–1.12)	1.06 (1.05–1.08)	1.12 (1.09–1.14)	0.86 (0.84–0.88)
	15	1.68 (1.66–1.70)	1.35 (1.33–1.38)	2.07 (2.02–2.11)	1.39 (1.34–1.43)
**Sex**	Female ®	1	1	1	1
	Male	1.24 (1.22–1.25)	1.37 (1.36–1.39)	1.31 (1.29–1.33)	1.42 (1.39–1.45)
**Grade**	1st intermediate ®	1	1	1	1
	2nd intermediate	0.96 (0.94–0.97)	0.83 (0.81–0.84)	1.24 (1.21–1.27)	1.10 (1.07–1.13)
	3rd intermediate	1.54 (1.52–1.56)	1.06 (1.04–1.08)	2.02 (1.97–2.06)	1.26 (1.22–1.30)
**Current cigarette smoker**	No ®	1	1	1	1
	Yes	15.1 (14.7–15.5)	9.8 (9.5–10.0)	25.9 (25.4–26.6)	15.5 (15.1–16.0)
**Exposure to family secondhand smoke**	No ®	1	1	1	1
	Yes	3.63 (3.59–3.67)	3.02 (2.98–3.06)	5.02 (4.94–5.10)	3.51 (3.45–3.58)
**Weekly pocket money (SAR)**	<30 ®	1	1	1	1
	≥30	1.38 (1.36–1.39)	1.23 (1.22–1.25)	1.82 (1.79–1.85)	1.59 (1.57–1.62)
**Exposure to tobacco promotional marketing**	No ®	1	1	1	1
	Yes	2.73 (2.70–2.76)	2.23 (2.21–2.26)	3.59 (3.53–3.66)	2.64 (2.58–2.69)
**Exposure to anti-tobacco messages**	No ®	1	1	1	1
	Yes	1.37 (1.35–1.38)	1.18 (1.17–1.19)	1.52 (1.49–1.54)	1.28 (1.26–1.31)
**Taught in school**	No ®	1	1	1	1
	Yes	1.09 (1.08–1.11)	0.87 (0.86–0.89)	1.71 (1.15–1.19)	0.85 (0.83–0.87)

AOR: adjusted odds ratio. Odds ratios are weighted to account for complex survey design.

* All statistically significant, p<0.05.

® Reference categories. SAR: 100 Saudi Arabian Riyals about US$27.

Adolescents who currently smoked cigarettes had almost 10 times the odds of ever using e-cigarettes (AOR=9.8; 95% CI: 9.5–10.0) and approximately 16 times the odds of currently using e-cigarettes (AOR=15.5; 95% CI: 15.1–16.0) compared with non-smokers. Exposure to SHS in the household was associated with approximately threefold higher odds of ever using e-cigarettes (AOR=3.02; 95% CI: 2.98–3.06) and over 3 times the odds of being current e-cigarette users (AOR=3.51; 95% CI: 3.45–3.58) compared with no exposure to SHS.

Adolescents receiving weekly allowances ≥30 SAR had higher odds of ever using e-cigarettes (AOR=1.24; 95% CI: 1.22–1.25) and being current e-cigarette users (AOR=1.59; 95% CI: 1.57–1.62) compared to those receiving weekly allowances <30 SAR.

Exposure to tobacco promotional marketing was associated with over 2 times the odds of being ever e-cigarette users (AOR=2.23; 95% CI: 2.21–2.26) and 2.76 times the odds of being current e-cigarette users (AOR=2.64; 95% CI: 2.58–2.69) compared with no exposure. Moreover, adolescents exposed to anti-tobacco messages had higher odds of current e-cigarette use (AOR=1.28; 95% CI: 1.26–1.31) compared to those unexposed (Table 3).

## DISCUSSION

This national study leveraged data from the GYTS 2022 to reveal the demographic characteristics, habits, and factors prevalent and associated with e-cigarette use among adolescents in Saudi Arabia. Results showed that 14.2% of adolescents had ever used e-cigarettes and that 5.3% were current users. E-cigarette use was more prevalent among older adolescents, males, and those with higher weekly allowance. Additionally, e-cigarette use among adolescents was associated with cigarette smoking, exposure to SHS, and exposure to tobacco promotional marketing. Unexpectedly, exposure to anti-tobacco messaging was also associated with a higher likelihood of e-cigarette use in this age group.

The alarming increase in e-cigarette use among adolescents is not solely a problem in Saudi Arabia, but rather worldwide. Multiple reports from across Europe^24^, the United States^25^, Asia^26^, and Africa^27^ highlight a growing trend of e-cigarette use among the youth. These reports even suggest that adolescent e-cigarette use surpasses adult use. However, this finding has not been definitively established in Saudi Arabia, given that the latest adult surveillance data (from 2019)^28^ showed low reported ever use (3.1%) and current use (0.8%) of e-cigarettes among adults; this result is still believed to be higher than that of adolescent use. Nevertheless, the rise in adolescent e-cigarette use is concerning, particularly because it seems to involve first-time use of e-cigarettes as an emerging tobacco product rather than necessarily transitioning from traditional tobacco products^29^.

Our study highlights that exposure to SHS and tobacco marketing were significantly associated with both ever and current e-cigarette use among adolescents. Saudi Arabia’s anti-smoking law^30^ aligns with the World Health Organization Framework Convention on Tobacco Control^31^ by protecting minors from exposure in designated public spaces and prohibiting tobacco advertising; however, enforcement gaps seem to persist, given the emergence of tobacco products such as e-cigarettes. Enforcement efforts must be strengthened to safeguard adolescents from these harmful influences.

The high prevalence of dual use observed in our study raises significant public health concerns. This pattern aligns with emerging evidence that e-cigarette use during adolescence often leads to dual use rather than serving as a replacement for conventional cigarettes^12^. Dual use is concerning as it may result in cumulative harm. The high dual use rates observed may partly reflect the current regulatory environment in Saudi Arabia. While the country’s anti-smoking law^30^ includes provisions for tobacco control, the emergence of e-cigarettes has created new regulatory challenges. Two critical gaps exist: the lack of restrictions on e-cigarette marketing through social media platforms heavily used by adolescents, and the absence of regulations on youth-appealing flavors. These regulatory gaps, combined with aggressive digital marketing and flavored product availability, likely contribute to the high dual use rates.

Our study found that adolescents exposed to anti-tobacco messaging were significantly more likely to be both ever and current e-cigarette users. This seemingly counterintuitive finding can be explained through several mechanisms. First, adolescence represents a developmental stage characterized by increased risk-taking and resistance to authority. Health warnings during this period may trigger psychological reactance, whereby attempts to discourage behavior paradoxically increase curiosity and experimentation with prohibited substances, including e-cigarettes^32^. Second, current users may be more attentive to and therefore more likely to recall anti-tobacco messages due to the messages’ personal relevance, a phenomenon documented in previous research^33^. This finding should not discourage efforts in anti-smoking and vaping prevention campaigns. Instead, it highlights the need for a more comprehensive approach. Engaging adolescents in campaign design and implementation with the use of tailored health communication strategies can help mitigate these potential unintended consequences of campaign exposure.

### Limitations

While this is the first nationally representative study in Saudi Arabia, it has several limitations. Self-reported data may introduce biases such as recall or social desirability bias, which could impact the accuracy of our estimates regarding e-cigarette use and its associated factors. Moreover, causal inferences could not be established because of the study’s cross-sectional design. The data were also inadequate to assess the frequency and specific characteristics of e-cigarette use, preventing a thorough analysis of its associations with other factors. The results are representative of Saudi Arabia; however, caution should be exercised when generalizing these findings to other countries.

### Implications

This study highlights the urgent need for public health interventions to curb e-cigarette use among Saudi Arabian adolescents:

- Older adolescents and males show high e-cigarette use, necessitating targeted, sex-specific prevention programs.
- Tobacco marketing has a significant impact on youth; hence, stricter regulations on advertising and promotions are needed.
- School-based anti-tobacco programs should be enhanced because current efforts seem inadequate.
- Parents should manage and monitor adolescents’ allowances to prevent e-cigarette purchases.

## CONCLUSIONS

This national study reveals that e-cigarette use is more prevalent among older adolescents, males, and those with higher weekly allowance. Additionally, e-cigarette use is associated with adolescents who use combustible cigarettes, are exposed to SHS, or encounter tobacco promotional marketing. Our findings collectively indicate a troubling association of exposure to both anti-tobacco and pro-tobacco messaging with adolescent vaping behaviors. They highlight the intricate and influential role of media in shaping youth vaping habits, suggesting that pro-tobacco messages have a greater influence on e-cigarette smoking prevalence than anti-tobacco messages and interventions. Developing and enforcing stricter tobacco control measures and implementing tailored messaging campaigns targeting adolescents are strongly recommended to protect this vulnerable population.

## Data Availability

The data supporting this research are available from the authors on reasonable request, with the permission of the General Directorate of Research and Studies, Ministry of Health, Riyadh, Saudi Arabia.
